# High calories but not fat content of lard-based diet contribute to impaired mitochondrial oxidative phosphorylation in C57BL/6J mice heart

**DOI:** 10.1371/journal.pone.0217045

**Published:** 2019-07-02

**Authors:** Larisa Emelyanova, Anna Boukatina, Cheryl Myers, Janice Oyarzo, Joseph Lustgarten, Yang Shi, Arshad Jahangir

**Affiliations:** 1 Center for Integrative Research on Cardiovascular Aging, Aurora St. Luke’s Medical Center, Aurora Health Care, Milwaukee, Wisconsin, United States of America; 2 Mayo Clinic, Scottsdale, Arizona, United States of America; 3 Aurora Cardiovascular Services, Aurora Health Care, Milwaukee, Wisconsin, United States of America; University of Illinois, UNITED STATES

## Abstract

**Purpose:**

High calorie intake leads to obesity, a global socio-economic and health problem, reaching epidemic proportion in children and adolescents. Saturated and monounsaturated fatty acids from animal (lard) fat are major components of the western-pattern diet and its regular consumption leads to obesity, a risk factor for cardiovascular disease. However, no clear evidence exists whether consumption of diet rich in saturated (SFAs) and monounsaturated (MUFAs) fatty acids has detrimental effects on cardiac structure and energetics primarily due to excessive calories. We, therefore, sought to determine the impact of high calories versus fat content in diet on cardiac structure and mitochondrial energetics.

**Methods:**

Six-week-old C57BL/6J mice were fed with high calorie, high lard fat-based diet (60% fat, HFD), high-calorie and low lard fat-based diet (10% fat, LFD), and lower-calorie and fat diet (standard chow, 12% fat, SCD) for 10 weeks.

**Results:**

The HFD- and LFD-fed mice had higher body weight, ventricular mass and thickness of posterior and septal wall with increased cardiomyocytes diameter compared to the SCD-fed mice. These changes were associated with a reduction in the mitochondrial oxidative phosphorylation (OXPHOS) complexes I and III activity compared to the SCD-fed mice without significant differences between the HFD- and LFD-fed animals. The HFD-fed animals had higher level of malondialdehyde (MDA) than LFD and SCD-fed mice.

**Conclusions:**

We assume that changes in cardiac morphology and selective reduction of the OXPHOS complexes activity observed in the HFD- and LFD-fed mice might be related to excessive calories with additional effect of fat content on oxidative stress.

## Introduction

High calorie intake leads to obesity, an emerging global socio-economic and health problem, reaching epidemic proportion in children and adolescents [1–6]. Animal (lard) fat is commonly used as a cooking fat or shortening in many cuisines and a major ingredient in sausages and various pastries consumed by children and adults. Saturated and monounsaturated fatty acids from animal (lard) fat are major components of the western pattern diet and its regular consumption leads to obesity that compromises cardiovascular health [7,8]. Studies of dietary fat composition still remain one of the most inscrutable and contradictory research fields in biology and nutrition due to complexity of structure and diversity of function of fatty acids in biological cell and tissue [9]. It has been shown that the type, source and composition of a diet strongly impact heart bioenergetics altering cardiac function [10–14] through changes in gene expression, metabolism, fatty acid composition and cholesterol content of cell membranes that alter ion fluxes, mitochondrial energetics, oxidative stress and conformation and function of membrane receptors or membrane-bound enzymes and transporters [15–20]. Fatty acids are the main metabolic substrates for the heart; however, excessive fat consumption may induce mitochondrial failure and activates molecular mechanisms of cardiac remodeling [11, 21]. Studies on animals and cell culture have provided mechanistic insight into the detrimental cellular effects of saturated fatty acids (SFAs), particularly palmitate and stearate, supporting the concept that SFAs are toxic to normal cellular processes [22, 23]. Palmitate has been shown to induce apoptosis, activation of stress-associated protein kinases, and protein oxidative stress in ventricular cardiomyocytes [22]. An obesogenic diet based on milk fat rich with C14 induced cardiac dysfunction, both gross and cellular hypertrophy, and increased autophagy in hearts of C57BL/6J mice [24]. An elevated intake of the n-6 polyunsaturated fatty acids (PUFAs) alone such as linoleic acid has been shown to enhance negative pro-inflammatory, pro-thrombotic and pro-arrhythmogenic effect [25–27]. The combination of n-3 and n-6 PUFA, compared with n-6 PUFA alone, appears to have different cardiac effects. Replacement of SFAs with a diet of mixed n-3 and n-6 PUFAs reduces risk for coronary heart disease, while intake of only n-6 PUFA increases the risk [28, 29]. In contrast, other reports found no evidence of HFDs enriched with SFAs on cardiac dysfunction and energetic impairment [30, 31]. In addition, some studies demonstrated that high fat diet can be potentially cardioprotective [32, 33]. The discrepancies in the literature reports could be explained by different experimental models, strains of animals, types of diets used for the studies, age or sex of animals. In addition, many studies overlook calorie value of the diets and calorie intake by the animals. No clear evidence exists on the effect of excessive calorie intake on cardiac structure and energetics.

The purpose of this study was, therefore, to compare the effect of the lard fat-based high calorie, high fat diet (HFD) vs. high calorie but low fat diet (LFD) and low calorie, low fat standard chow diet (SCD) on cardiac structure, functional activity of cardiac mitochondrial oxidative phosphorylation (OXPHOS) and oxidative stress.

## Materials and methods

### Animal care

All animal protocols were approved by the Institutional Animal Care and Use Committee of Mayo Clinic (Scottsdale, AZ) All ethical and animal care issues were adequately addressed. One-month-old male C57BL/6J mice were purchased from Harlan Laboratories (Madison, WI). Animals were housed in a temperature-controlled room (22 ± 1°C) and constant humidity conditions on a 12-h light/dark cycle and were fed *ad libitum* in Mayo Clinic Arizona Animal Facility. All mice were fed low calorie standard chow diet (SCD) post weaning until 5 weeks of age. After one week of acclimatization, the mice (6 weeks old) were randomly switched to high calorie, low lard fat-based diet (LFD, 4057 kcal/g, 10% kcal from fat, OpenSource Diet, D12450B; n = 7) and high calorie, high lard fat-based diet (HFD, 4057 kcal/g, 60% kcal% from fat, OpenSource Diet, D12492; n = 7) or continued on SCD (3040 kcal/g, 12% kcal from fat, PicoLab Rodent Diet 20, 5053, n = 7) for a further 10 weeks. After 5 weeks in the diets, mice were individually housed to monitor food intake. Terminal endpoint harvest of the heart was done in the fed-state in the morning.

### Heart isolation

After carbon dioxide (CO_2_) euthanasia, animals were weighed, hearts were excised; atria and blood were carefully removed. Ventricles were weighed; a portion sectioned for histology and the remaining tissue was immediately frozen in liquid nitrogen and kept at -80°C until further experiments.

### Histology

Coronal sections (1.5 mm thickness) were taken from the middle part of the hearts (at the level of the tip of the papillary muscle) using a mouse heart slicer (Zivic Instruments, model HSMS005-1). Slices were fixed in formalin solution (10% neutral buffered formalin and 0.12 M NaCl) in a refrigerator for at least 24 hours. Serial 5-micron paraffin sections were stained with hematoxylin and eosin (H&E) or Masson’s trichrome. Slides stained with H&E were analyzed at 40x magnification to calculate cardiomyocytes diameter. Twenty-five longitudinally sectioned cells in nucleus region from each slide were evaluated (125 cells/per mouse). Septal, posterior wall thickness and left ventricular internal diameter were calculated at 10x magnification using Adobe Photoshop software (Adobe Photoshop CS6). For collagen detection, Masson’s trichrome stained slides were assessed at 10x magnification. Three images per slide from identical areas were assessed for percent (%) area that was positive for Masson’s staining, using NIH Image J software.

### Left ventricular mass estimation

Left ventricular mass (LVM) was quantified according to the equation:

LVM=1.05* [(IVSD + LVD +LVPWD)³ – (LVD)³]

where 1.05 is a factor of myocardium-specific density, IVSD and LVPWD are left ventricular wall dimensions (interventricular septum wall and posterior wall thickness, respectively), and LVD is a left ventricular chamber diameter [34, 35].

#### Preparation of homogenate

Frozen heart tissue was homogenized in a cold (2–4°C) medium containing 100 mM KCl, 5 mM MgCl_2_, 2 mM EGTA, 50 mM Tris-HCl (pH 7.5) with a glass/glass homogenizer (1:30 w/v). Homogenate was centrifuged at 650xg for 10 min and filtered through polypropylene mesh (mesh opening size 0.125 mm). Aliquots of the supernatant were then used for the biochemical analysis. The protein concentration was determined in the supernatant using Bio-Rad DC Protein Assay.

### Assessment of mitochondrial enzyme activities

The measurement of the functional activity of OXPHOS complexes was performed spectrophotometrically at 30°C as described by Barrientos with modifications [36, 37].

NADH—Ubiquinone Oxidoreductase (Complex I) activity was assayed as a decrease in absorbance at 340 nm by following the oxidation of NADH (0.2 mM). The incubation medium contained 10 mM Tris (pH 8.0), 1 mg/ml BSA, 0.5 mM azide Na, 4 μg/ml antimycin A, and 80 μM DB (2,6-dichlorphenolindophenol by 2,3-dimethoxy-5-methyl-6-n-decyl-1,4-benzoquinone). The activity of complex I was determined using the rotenone (4 μM) sensitive rate and extinction coefficient of 6.22 mM^-1^cm^-1^ for NADH was applied for calculation of specific activity.

NADH—Cytochrome *c* Oxidoreductase (Complex I-III) activity was performed at 340 nm following the increase in absorbance resulting from the reduction of cytochrome *c*. The reaction medium contained 10 mM Tris (pH 8.0), 1 mg/ml BSA, 80 μM oxidized cytochrome *c*, 0.5 mM azide Na. The reaction was initiated by addition of 0.8 mM NADH. The specific rotenone-sensitive rate was calculated with an extinction coefficient of 6.22 mM^-1^cm^-1^ for NADH.

Succinate—Ubiquinone Oxidoreductase (Complex II) activity was determined by following the reduction of DCPIP (2,6-dichlorphenolindophenol) by DB at 600 nm. The reaction mixture contained 10 mM KH_2_PO_4_ (pH 7.8), 2 mM EDTA, and 1 mg/ml BSA, 80 μM DCPIP, 4 μM rotenone, 4 μg/ml antimycin A, 0.5 mM azide Na, 0.2 mM ATP, 10 mM succinate. The reaction was initiated by the addition of 80 μM DB. The activity was expressed by using 1 mM TTFA (2 –thenoyltrifluoroacetone), an inhibitor of complex II.

Succinate—Cytochrome *c* Oxidoreductase (Complex II-III) activity was assessed at 550 nm following the increase in absorbance resulting from the reduction of cytochrome *c*. The reaction medium contained 10 mM KH_2_PO_4_ (pH 7.8), 2 mM EDTA, and 1 mg/ml BSA, 4 μM rotenone, 4 mM azide Na, 0.2 mM ATP, 10 mM succinate. The reaction was started by addition of 80 μM oxidized cytochrome *c*. The activity of complex II was measured by using 1 mM TTFA sensitive rate.

Ubiquinol–Cytochrome *c* Oxidoreductase (Complex III) activity was assessed at 550 nm by monitoring the rate of reduction of cytochrome *c* by DBH_2_ (reduced DB). The reaction was initiated with the addition of oxidized cytochrome *c* in the buffer containing 10 mM KH_2_PO_4_ (pH 7.8), 2 mM EDTA, 1 mg/ml BSA, 80 μM DBH_2_, 4 μM rotenone, 0.5 mM azide Na, 0.2 mM ATP. The specific activity of complex III was calculated by subtracting the rate in the presence of 4 μg/ml antimycin A.

Cytochrome *c* oxidase (Complex IV) activity was measured by following the oxidation of reduced cytochrome *c* (40 μM) as a decrease in absorbance at 550 nm. The reaction buffer contained 10 mM KH_2_PO_4_ (pH 6.5), 250 mM sucrose, 1 mg/ml BSA, 2.5 mM *n*-Dodecyl β-D-maltoside.

The activities of complex II, III, I-III, II-III, and IV were calculated by using 19.1 mM^-1^cm^-1^ extinction coefficient.

F_1_F_0_-ATP synthase (Complex V) was assessed using lactate dehydrogenase and pyruvate kinase as coupling enzymes at 340 nm following decrease of absorbance as a result of the reduction of NADH. The medium contained 50 mM HEPES (pH 8.0), 5 mM MgSO_4,_ 4 μM rotenone, 4 μg/ml antimycin A, 0.35 mM NADH, 5 mM phosphoenolpyruvate, 50 μg/ml pyruvate kinase, 50 μg/ml lactate dehydrogenase, 1 mM ATP. The specific oligomycin A-sensitive rate was calculated with an extinction coefficient of 6.22 mM^-1^cm^-1^ for NADH.

Citrate Synthase (CS) activity was measured in media containing 100 mM KCl, 5 mM MgCl_2_, 2 mM EGTA and 50 mM Tris-HCl (pH 7.5). Reaction media was supplemented with 10 mM 5,5’-dithiobis-2-nitrobenzoic acid, 30 mM acetyl-CoA, and 10 mM oxaloacetic acid. The extinction coefficient for 5-thio-2-nitrobenzoic acid (TNB) was 13.6 mM^-1^cm^-1^ at 412 nm. The CS functional activity was expressed as nmol TNB/min/mg protein and used for normalization of OXPHOS complex activity.

The functional activities of the OXPHOS complexes were normalized to CS activity as described previously [36].

### Lipid peroxidation assay

Lipid peroxidation was assessed by measuring malondialdehyde (MDA) in supernatant as described by Buego and Aust [38]. MDA reacting with thiobarbituric acid results in the formation of a red substance with maximum absorption at 535 nm. MDA concentration of the sample was calculated using an extinction coefficient of 1.56×10 M ^-1^cm^-1^.

### Chemicals

Chemicals for biochemical and histological assessment were purchased from Sigma-Aldrich (St. Louis, MO, USA) and Thermo Fisher Scientific (Waltham, MA, USA).

### Statistical analysis

The sample size (n = 7) was calculated using the size of the effect and the estimate of the population standard deviation of the estimated parameter (http://www.biomath.info). Results were presented as a mean ± standard deviation (SD). The analyses were carried out using SigmaPlot 13.0 software. Grouped data were first analyzed for normality using the Shapiro-Wilk test. If the data failed normal distribution assumption, the Kruskal Wallis test was applied to compare the difference between the groups. One-way analysis of variance (ANOVA) with all pairwise multiple comparison procedures (Holm-Sidak method) was used to assess the significance of differences between normally distributed groups. Differences at p<0.05 were considered to be statistically significant.

## Results

### Composition of diets used in the study

We compared caloric composition, percentage contribution of calories from proteins, carbohydrates and fat, and fatty acids profile of the diets provided by nutritional companies (Tables 1 and 2). The SCD contained 11.9% of total calories from fat with 19.5%, 21%, and 59.5% of those calories originating from saturated, monounsaturated and polyunsaturated fatty acids, respectively. The LFD contained 10% of total calories from fat with 23.5%, 29.7% and 46.8% of those arising from saturated, monounsaturated and polyunsaturated fatty acids, respectively. The HFD contained 60% of total calories from fat with equal parts of SFA (32.2%), MUFA (35.9%), and PUFA (31.9%). In addition of different percentages of fat, there was no difference in the calories from protein (Table 1). Both the SCD and LFD had similar percentages of total calories from carbohydrates, which was higher than in the HFD (Table 1). The total amount of calories in the SCD was less than in the LFD and HFD diets.

**Table 1:** Diet composition based on caloric distribution.

	SCD	LFD	HFD
Protein, kcal %	23.6	20.0	20.0
Carbohydrates, kcal %	64.5	70.0	20.0
Fat, kcal %	11.9	10.0	60.0
Total energy, kcal/g	3040	4057	4057

Composition of individual polyunsaturated fatty acids expressed as percentage of total polyunsaturated fatty acids content. HFD, high fat diet; LFD, low lard fat diet; SCD, standard chow diet.

**Table 2:** Diet fatty acids composition.

Fatty acids	SCD	LFD	HFD
C10, Capric	0.0	0.0	0.2
C12, Lauric	0.0	0.0	0.2
C14, Myristic	0.6	0.3	2.8
C15, Pentadecanoic	0.0	0.0	0.2
C16, Palmitic	14.9	6.4	49.9
C16:1, Palmitoleic, n-9	0.0	0.3	3.4
C17, Margaric	0.0	0.1	0.9
C18, Stearic	3.2	3.1	26.9
C18:1, Oleic, n-9	19.0	12.3	86.3
C18:1, Vaccenic, n-7	1.4	0.0	0.0
C18:2, Linoleic, n-6	49.7	17.8	72.7
C18:3, Linolenic, n-3	6.2	2.1	5.1
C18:4, Octadecatetraenoic, n-3	0.2	0.0	0.0
C20, Arachidic	0.3	0.1	0.5
C20:1, Eicosenoic, n-9	0.2	0.2	1.6
C20:2, Eicosadienoic, n-6	0.1	0.2	2.0
C20:3, Homo-Gamma-Linolenic, n-6	0.0	0.0	0.3
C20:4, Arachidonic, n-6	0.1	0.1	0.7
C20:5, Eicosapentaenoic, n-3	1.0	0.0	0.0
C22, Behenic	0.3	0.1	0.1
C22:5, Docosapentaenoic, n-3	0.2	0.0	0.2
C22:6, Docosahexaenoic, n-3	1.1	0.0	0.0
C24:1, Nervonic, n-9	0.1	0.0	0.0
Total, %	98.6	43.1	254.0
Saturated (%)	19.5	23.5	32.2
Monounsaturated (%)	21.0	29.7	35.9
Polyunsaturated (%)	59.5	46.8	31.9
n-6	49.9	17.9	73.7
n-3	8.7	2.1	5.3
n-6/n-3 ratio	5.7	8.4	13.9
Cholesterol, mg/kg	142.0	51.6	300.8

Analysis of fatty acid composition in the diets revealed that palmitic (C16), oleic (C18:1, n-9), linoleic (C18:2, n-6), and linolenic (C18:3, n-3) were generally predominant in all three diets with some variety between the diets (Table 2). Stearic acid (C18) was highly abundant in the HFD and less present in the SCD and LFD. The HFD was richer in myristic (C14), palmitoleic (C16:1, n-9), margaric (C17), eicosenoic (C20:1, n-9), eicosadienoic (C20:2), and arachidonic (C20:4, n-6) acids. The SCD had more PUFAs in forms of eicosapentaenoic (C20:5, n-3) and docosahexaenoic (C22:6, n-3). The SCD had lower n-6/n-3 ratio (Table 2). The HFD had a significantly higher level of cholesterol content than the SCD and LFD (Table 2).

### Body mass and morphometric characteristics of heart

**Fig 1:**
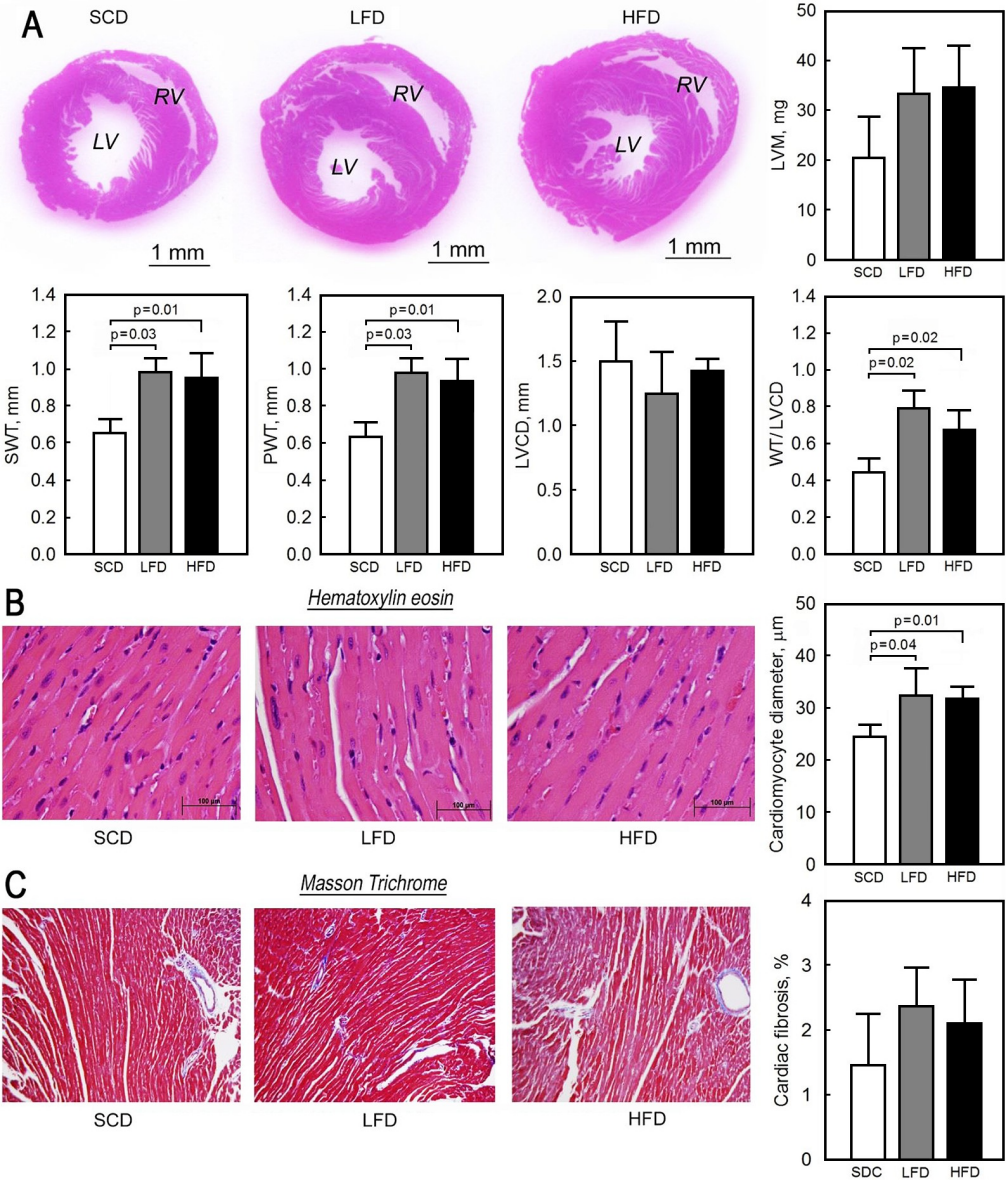
The effect of low lard fat-based (LFD), high lard fat-based (HFD) and standard chow (SCD) diets on cardiac structure and histology. (A) Hematoxylin and eosin-stained ventricle mid-region heart cross sections and quantitative morphometry of septal (SWT) and posterior (PWT) wall thickness, left ventricular chamber diameter (LVCD), ratio of left ventricular wall thickness (WT) to LVCD, and left ventricular mass (LVM) from the LFD-, HFD- and SCD-fed mice. (B) Representative histological sections stained with hematoxylin and eosin (40x) and quantification of cardiomyocyte diameter. (C) Representative slides stained with Masson’s trichrome (10x); fibrosis was evaluated as a percentage of blue-stained pixels (collagenous tissue) to the sum of blue and red pixels (total tissue area) x 100% using Image J software. Data presented as mean ± SD, n = 7 for the SCD, n = 7 for the LFD, and n = 7 for the HFD. LV–left ventricular; RV–right ventricular.

After 10 weeks of feeding, the overall weight gain in the mice fed with the lard fat-based diets was 1.6- and 3-times higher in the LFD and HFD groups, respectively, than in the SCD animals. The SCD mice calorie intake was significantly less compared to the LFD and HFD mice (Table 3). However, we did not observe statistically significant changes in the ratio of the ventricles weight to the body weight (Table 3). This was associated with significant increases in the thickness of the left ventricular posterior wall (0.64±0.08 mm in SCD vs. 0.98±0.07 mm in LFD and 0.93±0.12 mm in HFD, p = 0.01) and septal wall (0.65±0.07 mm in SCD vs. 0.99±0.07 mm in LFD and 0.95±0.13 mm in HFD, p = 0.01) in mice fed with the LFD and HFD (Fig 1A). The overall left ventricular mass was higher in the LFD (33.36±9.08 mg) and HFD (34.69±8.32 mg) mice compared to the SCD (20.54±8.17 mg) animals but statistically non-significant (p = 0.15) and the pattern of hypertrophy appeared to be concentric with a small chamber size (Fig 1A) relative to the ventricular wall thickness. The left ventricular wall thickness normalized to the chamber diameter was significantly increased in mice fed with the LFD (0.79±0.02) and HFD (0.68±0.05) compared to the SCD (0.45±0.04, p = 0.02)-fed animals (Fig 1A). The histological analysis also revealed a hypertrophic phenotype at the cellular level with a 24% increase in cardiomyocytes diameter in the LFD (32.32±5.22 nm) and HFD (31.83±2.25 nm) mice compared to the SCD mice (24.49±2.31 nm, p = 0.03) with a normal linear arrangement of myofibrils in ventricles in all three groups of animals (Fig 1B). The degree of interstitial and perivascular fibrosis was not different between the three groups (Fig 1C).

**Table 3:** Biometric data and calorie intake of mice fed with standard chow, low lard fat- and high lard fat-based diets.

	SCD	LFD	HFD
Body weight, g (at 6 weeks)	18.00 ± 0.50	18.28 ± 0.61	18.25 ± 0.56
Body weight, g (at 16 weeks)	26.50 ± 1.29	32.14 ± 2.87^a^	44.40 ± 6.08^a^^b^
Gain of body weight, g	8.50 ± 0.50	13.86 ± 2.32^a^	26.15 ± 3.81^a^^b^
Ventricle weight, g	0.12 ± 0.01	0.14 ± 0.02^a^	0.15 ± 0.01^a^
Ventricle weight/Body weight	0.0044 ± 0.0003	0.0044 ± 0.0006	0.0037 ± 0.0005
Calorie intake, kcal/mouse/day	12.99 ± 1.69	93.25 ± 2.78 ^a^	114.81 ±3.07 ^a^^b^

All data are presented as mean ± SD, n = 7 each group

^a^ p<0.05 vs SCD

^b^ p<0.05 vs LFD.

HFD, high fat diet; LFD, low lard fat diet; SCD, standard chow diet.

### Mitochondrial enzyme activities

**Fig 2:**
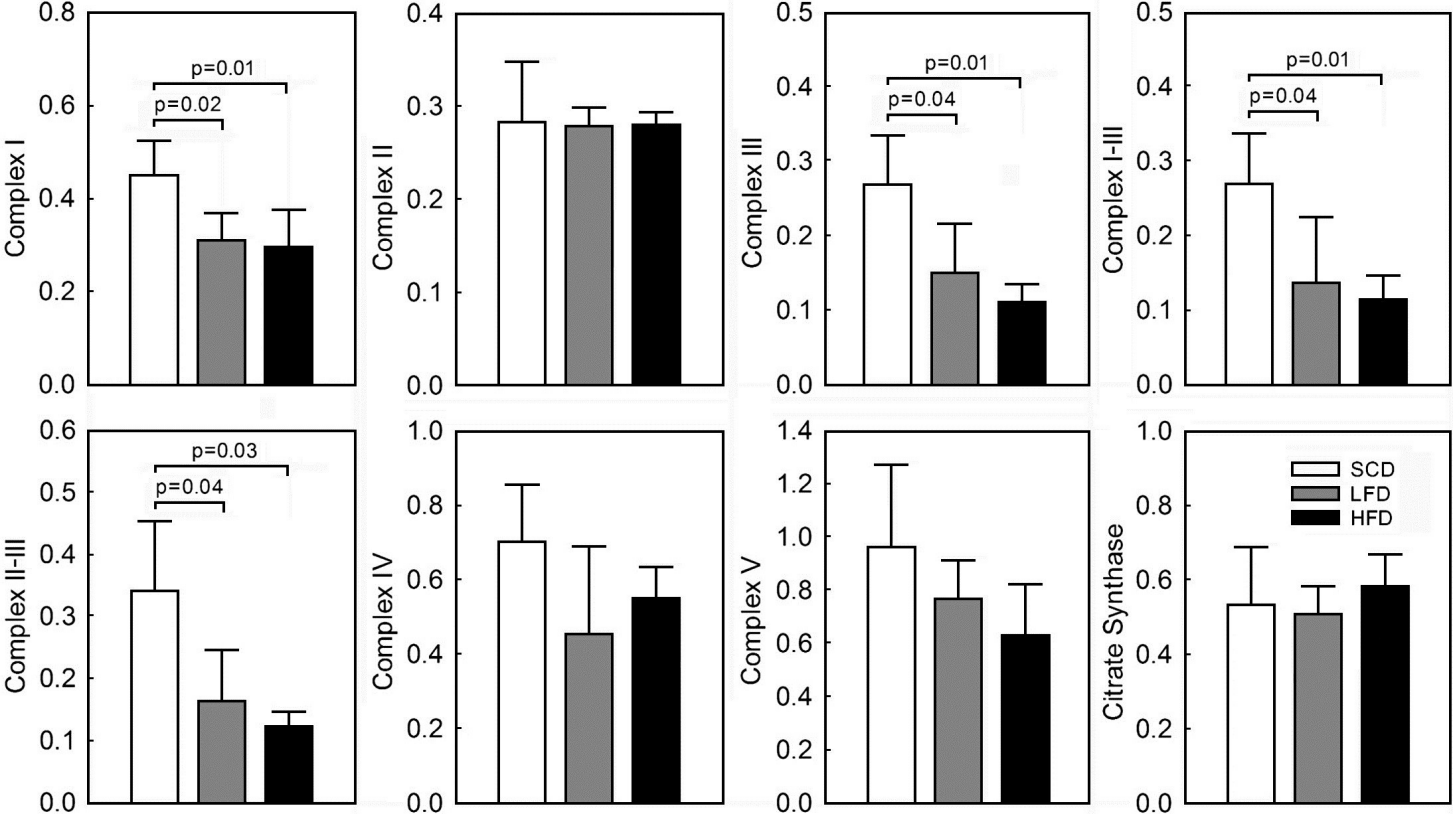
The effect of low lard fat-based (LFD), high lard fat-based (HFD) and standard chow (SCD) diets on activity of mitochondrial OXPHOS complexes and citrate synthase in ventricular tissue. The functional activity of OXPHOS complexes and citrate synthase was measured spectrophotometrically as described in Material and Methods and normalized to citrate synthase activity (nmol/min/citrate synthase activity). The citrate synthase activity was expressed as nmol TNB/min/mg protein. Data presented as mean ± SD, n = 7 for the SCD, n = 7 for the LFD, n = 7 for the HFD.

Functional activity of mitochondrial OXPHOS complexes in ventricular tissue from the mice fed with the LFD and HFD was selectively reduced compared to the SCD mice. There was no difference in functional activity of the complexes between the LFD and HFD animals (Fig 2). The overall activity of complex I was significantly reduced in the LFD (0.31±0.06 nmol/min/CS activity) and HFD (0.29±0.10 nmol/min/CS activity) mice compared to the SCD (0.45±0.07 nmol/min/CS activity, p = 0.02). There was no significant difference between the LFD and HFD animals in complex I activity (p = 0.69). Similarly, activity of complex III was significantly reduced in the LFD (0.16±0.07 nmol/min/CS activity) and HFD (0.11±0.02 nmol/min/CS activity) compared to the SCD (0.27±0.06 nmol/min/CS activity, p<0.01) mice. This was reflected in reduction of complex I-III (0.26±0.06 in SCD vs. 0.14±0.09 in LFD and 0.11±0.03 in HFD nmol/min/CS activity, p = 0.01) and II-III (0.33±0.1 in SCD vs. 0.16±0.08 in LFD and 0.12±0.02 in HFD nmol/min/CS activity, p<0.01) interactions. The activity of complex II (0.28±0.06 in SCD vs. 0.28±0.02 in LFD and 0.28±0.01 in HFD nmol/min/CS activity, p = 0.98), complex IV (0.72±0.16 in SCD vs. 0.49±0.24 in LFD and 0.55±0.09 in HFD nmol/min/CS activity, p = 0.14) and complex V (0.97±0.31 in SCD vs. 0.77±0.15 in LFD and 0.63±0.19 in HFD nmol/min/CS activity, p = 0.12) of OXPHOS was not significantly different between the groups. The CS activity was also not different between the three groups (0.54±0.16 in SCD vs. 0.51±0.08 in LFD and 0.58±0.09 in HFD μmol TNB/min/mg protein, p = 0.56) indicating no significant effect of the difference in calorie and fat content on mitochondrial content at 10 weeks of feeding.

### MDA concentration in ventricular tissue homogenate

**Fig 3:**
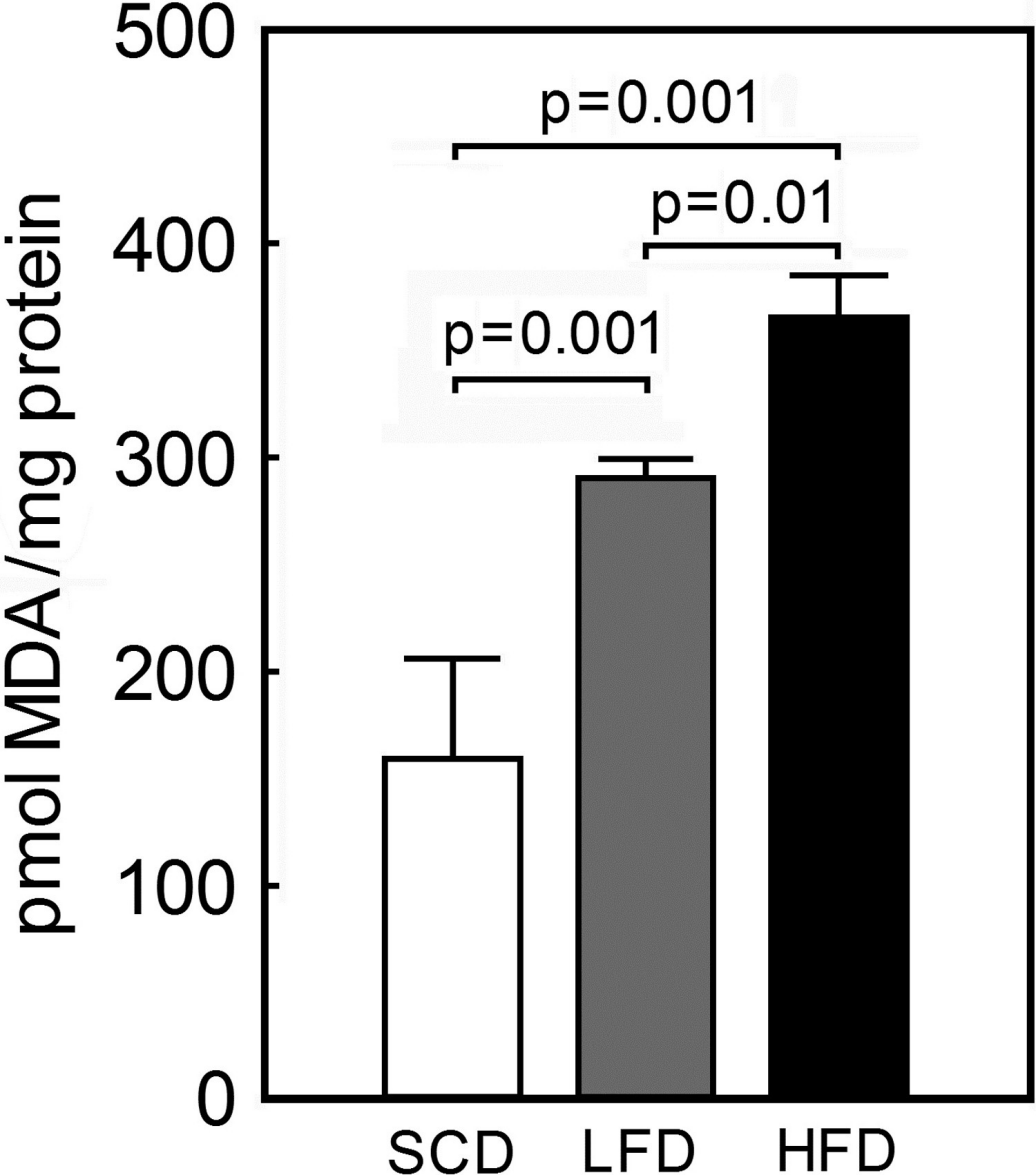
Malondialdehyde level in ventricular tissue from low lard fat-based (LFD), high lard fat-based (HFD) or standard chow (SCD) diet-fed mice. Malondialdehyde (MDA) concentration was determined by a thiobarbituric acid assay in ventricular tissue homogenates as described in Material and Methods. Data presented as mean ± SD, n = 5 for each group.

To determine the level of oxidative stress in the three groups of mice, the tissue level of MDA, a marker of lipid peroxidation was assessed in the ventricular tissue. Mice on the LFD and HFD had significantly higher MDA level compared to the SCD-fed mice (175.8±39.1 vs. 290.7±8.8 in LFD and 365.5±19.8 in HFD pmol/mg protein, p<0.001) (Fig 3) indicating higher oxidative stress in heart tissues of the animals fed high-calorie lard fat-based diets. The HFD-fed animals had significantly higher content of MDA compared to the isocaloric LFD-fed animals (p = 0.01) demonstrating that fat content additionally affects oxidative stress level.

## Discussion

The question we addressed in our study was to determine the impact of the excessive calories vs. fat content on cardiac structure, activities of OXPHOS complexes and oxidative stress. The main finding of our study is that young mice fed the LFD and HFD with high calorie content for 10 weeks developed left ventricular hypertrophy, which was associated with a selective reduction in the activity of cardiac mitochondrial OXPHOS complexes I and III and increased MDA production, a marker of oxidative stress, when compared to the mice fed the SCD. There was no significant difference in the extent of hypertrophy at the whole heart and cardiomyocyte level between the LFD and HFD fed isocaloric but different fat content diets. However, mice on the HFD diet had higher oxidative stress compared to the animals fed the LFD.

Cardiac hypertrophy is characterized with increased cardiomyocytes size, resulting in the thickening of the cardiac muscle wall [39]. Our study demonstrated that the consumption of the LFD and HFD diets resulted in significant increase in ventricular mass, as determined by the increases in left ventricular wall thickness (septal wall and posterior wall thickness), LVCD, and cardiomyocytes size, indicative of cardiac hypertrophy. These findings are suggestive of a deleterious effect of these two diets on the cardiac structure compared to the SCD. The LFD and HFD diets are different from the SCD mainly in the amount of calories, amount of fat (Table 1) and distribution of PUFAs, which was less in the two, lard fat-based diets derived from animal fat compared to the SCD (Table 2). The SCD had lower n-6/n-3 ratio compared to the LFD and HFD due to presence of eicosapentaenoic (C20:5, n-3) and docosahexaenoic (C22:6, n-3) acids in the diet. These PUFAs were not detected in the lard-fat based diets. The LFD had least amount of cholesterol vs. the SCD and HFD. This suggests that the cholesterol content in the diet does not differentially affect body weight gain, cardiac hypertrophy and mitochondrial function. In addition, mice on the SCD consumed fewer calories (calorie intake/mouse/day) compared to the LFD and HFD animals (Table 3). Thus, the overall impact of lard-based diets on cardiac structure, mitochondrial function and oxidative stress could be due to the excessive calories, fat composition, or both. A previous study on C57BL/6J mice demonstrated that long-term consumption of high-fat (45% calories from fat) non-obesogenic diet had no effect on the body weight and cardiac hypotrophy but independently of obesity-induced co-morbidities increased the vulnerability of cardiomyocytes to ischemia/reperfusion [40]. In contrast, Zeng et al. reported that feeding wild type (WT) and SIRT3 KO mice with HFD (60% calories from fat) accelerated obesity, cardiac hypotrophy and cardiac dysfunction that is similar to our results [41]. The use of HFD reduced SIRT3 level in WT mice, exacerbated oxidative stress in WT and SIRT3 KO mice that impaired hypoxia-inducible factor (HIF) signaling and reduced myocardial capillary density causing greater cardiac hypertrophy and dysfunction [41]. Another comparison of high calorie lard-based and low calorie hydrogenated vegetable-based diets (60% kcal vs. 16% kcal from fat) have demonstrated a deleterious effect of high lard fat-based diet on total energy intake, weight gain, visceral obesity and insulin resistance [42]. This is consistent with our results demonstrating ventricular hypertrophy only in animals fed the lard-based diets containing higher calories than the low calorie SCD. There was no additional effect of fat content on the cardiac hypertrophy and fibrosis or mitochondrial OXPHOS activity at 10 weeks between the HFD- and LFD-fed animals indicating that high calories might be primarily responsible for the deleterious effect of lard-fat based diets. However, a higher level of MDA, a marker of lipid peroxidation, was observed in the HFD compared to the LFD and SCD group of animals suggesting that the high fat content may be responsible for increase in oxidative stress. This may have additional deleterious effect on myocardial remodeling and fibrosis over the long term, beyond 10 weeks, which needs to be investigated further. Calligaris et al. fed C57BL/6J mice with HFD (60% of calories from fat) over a longer time (8, 12 and 16 months) and revealed cardiac remodeling at all time points [30]. This was confirmed by the overexpression of collagen types I and III at mRNA and protein levels. The cardiac remodeling was associated with thickening of the cardiac fibers and left ventricular wall that resulted in cardiac hypertrophy in their model [30]. Oxidative stress has been known to be implicated in the pathogenesis of cardiac remodeling including cardiac fibrosis through direct action or the involvement of reactive oxygen species in cytokine and growth factor signaling [43]. In addition, whether PUFAs in the SCD animals had a protective effect could not be determined in this study. Previous studies have shown that dietary lipid composition (saturated vs. unsaturated fatty acids) influenced cardiac structure and function. In rats fed with isocaloric lard fat-based vs. diets rich in vegetable oil from weaning to 18 months of age, cardiac hypertrophy, myocardial necrosis and collagen deposition was demonstrated mainly in animals fed with the lard fat-based diet [44]. The hypertrophy phenotype observed in this study is consistent with our findings in mice fed with the high calorie LFD and HFD diets for 10 weeks. However, we did not observe an increase in myocardial fibrosis, which could be due to a much shorter period of follow-up (10 vs. 70 plus weeks), difference in species or diets’ composition compared to their model [44]. Interestingly, Nguyen et al. fed FVB/N and C57BL/6J mice either western diet (45% fat, 60% SFAs), surwit diet (60% fat, 90% SFAs), milk fat-based diet (60% fat, 60% saturated or high fat diet (60% fat 32350) % SFAs) for 12 weeks and did not find changes in cardiac systolic function in both strains of mice [45]. However, C57BL/6J mice developed mild cardiac hypertrophy and displayed diastolic dysfunction if fed a high fat diet with equal parts of SFAs, MUFAs, and PUFAs. This study did not support the notion that diets high in SFAs would result in cardiac hypertrophy and dysfunction. Fish oil-based or n-3 polyunsaturated fatty acids-based diets have been previously shown to be protective against cardiac hypertrophy, remodeling, and contractile dysfunction in other animal models indicating that the source of fat could have a significant impact on cardiac function [46, 47].

Dietary imbalance and calorie excess have been demonstrated to lead to changes in mitochondrial energetics [48]. In our study, we observed a decrease in the functional activity of cardiac mitochondrial complexes I and III in mice fed with lard-fat based diet compared to the SCD mice. However, there were no significant changes in the activity of these complexes between the LFD and HFD animals. This is in the line with another report on C57BL/6J mice fed the lard-fat based diets (LFD and HFD) for 20 weeks that showed no changes in the activities of complex I and III in cardiac mitochondria from the LFD and HFD mice [49]. In addition, there were no abnormalities in the OXPHOS super complexes formation in the cardiac mitochondria despite the alterations in cardiac phospholipid acyl chains and mitochondrial membrane packing between the LFD and HFD groups. The authors suggested that mitochondrial acyl chain remodeling was not a major factor of impaired mitochondrial function in cardiac tissue. Surprisingly, liver mitochondrial complexes I and III activity were lower in the mice fed the HFD relative to the LFD and this was associated with impaired supercomplexes formation [49]. In our study, the precise mechanism underlying the reduced activity of mitochondrial OXPHOS complexes I and III in the LFD- and HFD-fed mice compared to SCD is not known but could be due to less supercomplex formation in the mice that consumed the lard fat-based diets. Future studies need to assess differences in supercomplex formation, mitochondrial oxygen consumption rate and coupling of oxidative phosphorylation and oxidant production between the animals that consumed the SCD and lard-fat based diets. Another potential mechanism underlying decreased activity in complex I and III could be related to cardiolipin deficiency in cardiomyocytes as the lard fat-based diet is mostly supplemented with palmitate [50] that may alter cardiolipin synthesis [51]. Cardiolipin is important for maintaining mitochondrial electron transport chain (ETC) integrity by helping to assemble and stabilize supramolecular complexes composed of individual complexes I, III and IV [52]. Supercomplexes, according to the solid-state model, allow high-efficiency electron flux through the ETC due to short diffusion distances between complexes; increasing efficiency of ATP synthesis and reducing electron leakage and production of reactive oxygen species (ROS) [53]. Deficiency in cardiolipin can result in impairment of electron transfer process in ETC and selective reduction in enzymatic activity of the individual complexes I and III due to its absolute requirement for proper functioning of these complexes [54].

The increase in oxidative stress has been shown to be involved in the pathogenesis of cardiovascular diseases related to metabolic syndrome and obesity [55]. The higher body mass index is associated with greater oxidative stress reflected by a higher level of oxidative biomarkers [56, 57]. Oxidative stress has often been linked to pathological morphological alterations in the heart as a response to high calorie diet or dietary lipid supplementation and plays a significant role in mediating myocardial hypertrophy [20,58,59]. In our study ventricular hypertrophy in the high-calorie fed mice was associated with incremental increase of MDA level, highest in the HFD-fed mice, intermediate in the LFD, and lowest in the SCD. Several potential mechanisms have been proposed to underlie the diet-induced oxidative stress in the heart including increased heart work and subsequent increase in mitochondrial superoxide production [12,20,60]; reduction in antioxidant reserves [55] and potential fat deposition in the myocardium [48,60]. Another potential mechanism for increased lipid peroxidation could be the enriched saturated fatty acid composition of the diet. Palmitate supplementation increases ceramide production *via de novo* synthesis pathway and accumulation of ceramide that triggers ROS production and increases oxidative stress due to activation of ROS-generating enzymes [61]. In addition, mitochondria themselves contribute to oxidative stress through the increase of ROS production as a result of dysfunctional ETC activity as previously described [62]. This is in line with our observation of the higher level of MDA in the LFD and HFD mice’s heart associated with reduced activity of complex I and III, two major sites of ROS production in the mitochondria.

One of the limitations of our study is that serum and cardiac tissue lipid profile in response to the different diets were not determined to assess if any discrepancies in tissue fatty acids would be critical in the pathogenesis of cardiac hypertrophy and mitochondrial dysfunction, which needs to be further investigated. Since food access was not restricted (*ad libitum*), mice on the high calorie lard fat-based diets (LFD and HFD) gained more weight compared to the SCD-fed animals due to increased caloric consumption. This assumption was also supported by increased calorie intake in mice fed the lard-fat based diets. The HFD–fed mice gained more weight than the LFD-fed mice, but there was no significant overall effect of lard fat amount on difference in cardiac morphology and energetics between the two groups of mice fed high but isocaloric diets. In support, another recent study on C57BL6 mice showed that prolonged *ad libitum* HFD feeding increased daily caloric intake in animals that correlated positively with weight gain [63]. Interestingly, no effect of the fat source on total daily calorie intake was observed. This finding suggested that the HFD–induced overeating and weight gain were driven by hedonic (palatability) signals that appeared to override homeostatic signals for body weight control [63]. Another limitation is that we did not compare the effect of another non-lard fat-based diet matching in calories with the LFD and HFD. In this regard, the adverse effect of the LFD and HFD on cardiac remodeling and energetics may not be attributed solely to the high calorie but fat composition (saturated vs. unsaturated and n-6/n-3 ratio) as well. This needs to be further investigated. In summary, we observed a significant impact of the high calorie lard fat-based diets (LFD and HFD) on cardiac remodeling, mitochondrial function and oxidative stress in the C57BL/6J mice compared to the low calorie SCD. The novelty of the study is the finding that the consumption of the lard fat-based diets identical in calorie amount but different in fat content (LFD vs. HFD) for 10 weeks did not affect the cardiac morphology and the activity of the mitochondrial OXPHOS complexes in the young C57BL/6J mice. However, increase of lard fat content in the diet resulted in additive increase of oxidative stress in the HFD-fed compared to LFD- and SCD-fed mice that over the long term could cause additional deleterious effect on myocardial remodeling and fibrosis. Further investigations into the mechanism underlying selective downregulation of the mitochondrial ETC by dietary interventions at a young age and whether age-related differences exist in the responsiveness of the heart [64] are warranted to identify targets to prevent mitochondrial dysfunction and adverse remodeling of the heart.

## Supporting information

S1 Dataset The file includes data on cardiac septal wall thickness (SWT), posterior wall thickness (PWT), left ventricular mass (LVM), left ventricular chamber diameter (LVCD), ration of left ventricular wall thickness (WT) to LVCD, cardiomyocyte diameter, cardiac fibrosis (Excel sheet—Fig 1); mitochondrial complexes (complex I, complex II, complex II, complex IV, complex V, complex I-III and complex II–III) and citrate synthase activity (Excel sheet—Fig 2); malondialdehyde (MDA) level (Excel sheet–Fig 3). (XLS)
